# YARS as an oncogenic protein that promotes gastric cancer progression through activating PI3K-Akt signaling

**DOI:** 10.1007/s00432-019-03115-7

**Published:** 2020-01-08

**Authors:** Cheng Zhang, Xiaoting Lin, Qian Zhao, Yakun Wang, Fangli Jiang, Congcong Ji, Yanyan Li, Jing Gao, Jian Li, Lin Shen

**Affiliations:** 1 Department of Gastrointestinal Oncology, Key Laboratory of Carcinogenesis and Translational Research (Ministry of Education/Beijing), Peking University Cancer Hospital and Institute, 52 Fucheng Road, Beijing, 100142 China; 2 Department of Medical Oncology, Cancer Hospital, The First Affiliated Hospital of Xiamen University, Xiamen, China; 3 Department of Clinical Medical, Fujian Medical University, Fuzhou, China; 4 Xiamen Key Laboratory of Antitumor Drug Transformation Research, The First Affiliated Hospital of Xiamen University, Xiamen, China; 5 National Cancer Center/National Clinical Research Center for Cancer/Cancer Hospital and Shenzhen Hospital, Chinese Academy of Medical Sciences and Peking Union Medical College, Shenzhen, 518116 China

**Keywords:** Gastric cancer, Oncogenic protein, YARS, PI3K-Akt, Homologous recombination

## Abstract

**Purpose:**

Members of the aaRS (aminoacyl-tRNA synthetase) family are proteins controlling the aminoacylation process, in which YARS (tyrosyl-tRNA synthetase) catalyzes the binding of tyrosine to its cognate tRNA and plays an important role in basic biosynthesis. Several studies have demonstrated the association between YARS mutation and certain developmental abnormalities/diseases, yet YARS’s linkage with cancer remains uncategorized. In this study, by combining in silico, in vitro, and in vivo studies, we explored the expressions and functions of YARS in gastric cancer (GC).

**Methods:**

We evaluated YARS’s distribution in tumor and paired normal tissues/specimens of GC by referring to large cohort online datasets and patient-derived tissue specimens. YARS-related changes were assessed by phenotypical/molecular experiments and RNA-sequencing analysis in GC cell lines harboring YARS knockdown or overexpression.

**Results:**

Both the transcript and protein levels of YARS were evidently higher in gastric cancer tissues than in paired normal tissues. YARS knockdown induced repressed proliferation and invasiveness, as well as enhanced apoptosis in GC cell lines, while abnormally upregulating YARS expression promoted gastric cancer growth in vivo. We inferred based on RNA-sequencing that YARS modulates multiple cancerous signaling pathways and proved through cellular experiments that YARS promoted GC progression, as well as homologous recombination by activating PI3K-Akt signaling.

**Conclusions:**

By revealing the existence of a YARS-PI3K-Akt signaling axis in gastric cancer, we discovered that tRNA synthetase YARS is a novel tumorigenic factor, characterized by its upregulation in tumor-derived specimens, as well as its functions in promoting gastric cancer progression.

**Electronic supplementary material:**

The online version of this article (10.1007/s00432-019-03115-7) contains supplementary material, which is available to authorized users.

## Introduction

Gastric cancer (GC) is one of the most prevalent malignancies around the world. With the rapid development of targeted therapy and immunotherapy, diagnosis and treatment of multiple types of cancer have been greatly improved, yet due to limited drug targets, as well as high heterogeneity, gastric cancer remains to be a major health problem (Siegel et al. 2017). Currently, HER2 (ERBB2)-targeted antibody Trastuzumab is the only first-line option for GC’s targeted treatment (Van Cutsem et al. 2016); for immunotherapy, the response rate for PD-1/PD-L1-based regimens is limited in GC population, while the choice of immune checkpoint inhibitors’ combination with chemotherapy or targeted agents were controversial (Lordick and Janjigian 2016). Therefore, uncovering novel druggable targets and mechanisms of gastric cancer progression is of urgent need for the development of precision medicine.

As housekeeping proteins prevalently found in all living organisms, members of the aaRS (aminoacyl-tRNA synthetase) family catalyze the binding of amino acids to tRNAs and translate the coding information from nucleic acids into amino acids, exerting fundamental roles in protein synthesis (Carter 1993). During evolution, tRNA synthetases incorporated multiple domains that expanded their functions beyond aminoacylation, allowing them to participate in diverse biological events, such as nuclear tRNA export, mitochondria RNA splicing, transcriptional and translational control, RNA maturation and retrovirus packaging (Cen et al. 2001; Ko et al. 2000, 2001; Liu et al. 2002; Sarkar et al. 1999). Among all aaRSs, YARS (tyrosyl-tRNA synthetase, aka TyrRS or YRS), the member that promotes the combination of tyrosine to its cognate tRNA, was recently potentiated to regulate intracellular signaling (Sun et al. 2017). Mutations of YARS have been prevalently reported to be associated with genetic diseases, including an autosomal dominant form of Charcot-Marie-Tooth (CMT) neuropathy (Fuchs et al. 2019), as well as a recently reported autosomal recessive multi-organ disease (characterized by failure to thrive, hypertriglyceridemia, developmental delay, and abnormalities/disorders in multiple organs) (Nowaczyk et al. 2017; Tracewska-Siemiatkowska et al. 2017).

On the other hand, the upregulation of aaRSs, including YARS, was observed in several types of cancer (Guo et al. 2010; Hsu et al. 2019), while polymorphisms in aaRS genes were reported to be associated with breast cancer risk (He et al. 2015). Emerging studies also implicated the involvement of aaRSs with multiple pathway networks through constituting the multi-tRNA synthetase complex (MSC) (Hyeon et al. 2019) and raised the possibility of aminoacyl-tRNA synthetases as therapeutic targets against autoimmune diseases, rare diseases, and even cancer (Kwon et al. 2019). Nevertheless, although abundantly exists and functions in all organisms, YARS’s actual linkage with cancer remains unspecified. In this study, by analyzing the omics datasets and performing experimental study with patient-derived specimens, cell lines, as well as animal models, we explored the functions and relevant mechanisms of YARS in gastric cancer.

## Materials and methods

### Datasets and patient specimens

We were free to download and use the oncomine database (https://www.oncomine.org/), the TCGA-gastric cancer dataset (*n* = 441, https://portal.gdc.cancer.gov/), the Gene Expression Omnibus database (https://www.ncbi.nlm.nih.gov/geo/), as well as a gastric cancer cohort (*n* = 84) previously testified by mass spectrum-based profiling/exome sequencing and published by our group (referred to as the MS data) (Ge et al. 2018).

Fourteen pairs of surgery resected tumor/adjacent samples were acquired from gastric cancer patients, collected by the department of Gastrointestinal oncology and department of Pathology, Peking University Cancer Hospital and Institute. The experimental applications of patient specimens were approved by the institutional ethics committee, Peking University Cancer Hospital and Institute. Written informed consents were obtained from all providers.

### Statistics and bioinformatic analysis

The diversity of expressions, copy number changes and mutations in different subgroups were compared with Student *t* test. YARS’s relevance with TMB (tumor mutation burden) was assessed with Pearson’s correlation analysis. YARS expression-related stratification was performed in a median-based manner. Prognostic (overall survival, OS; disease-free survival, DFS) changes were assessed with Kaplan–Meier analysis and log-rank test. Statistics were performed and formatted with SPSS 21.0, GraphPad 5.1 or Excel software. *p* < 0.05 was considered to be of statistical significance. Gene Set Enrichment Analysis (GSEA) was performed with GSEA software (v2.0.13) by adopting a permutation number of 1000. All gene sets were downloaded from the GSEA website (www.broadinstitute.org/gsea/).

### Cell lines, culturing, and transfection

Gastric mucosal epithelial cell line GES-1, gastric cancer cell lines HGC-27/AGS were purchased from ATCC (Manassas, VA). Gastric cancer cell line MGC-803 was purchased from Shanghai Institutes for Biological Sciences (Shanghai, China). RPMI-1640 medium (Invitrogen, Carlsbad, CA) supplemented with 10% fetal calf serum (Gibco BRL) and 1% penicillin plus streptomycin (HyClone, Logan, UT) was used for incubation. All cell lines were authenticated by short tandem repeat profiling. siRNAs, shRNAs, as well as plasmids were administered with the Lipofectamine 3000 reagent (Thermo Fisher, USA) for transfection.

### RNA-sequencing

Total RNA was collected from transfected cells using TRIzol method. Quality control was performed using an Agilent 2100 Bioanalyzer (Agilent Technologies) to ensure RNA integrity. Next generation RNA-sequencing was performed by Novogene (Beijing, China) using an Illumina HiSeq instrument (Illumina, San Diego, CA).

### Animal experiment

GC cells were digested with trypsin/EDTA (Gibco BRL) and resuspended to 2 × 10^7^ cells/mL with PBS. 100 μL of cell suspension was subcutaneously inoculated in the right flank of a 5-week-old female BALB/C nude mouse (Vital River Laboratories, Beijing, China). Mice weight and xenograft size were measured every 3 days. When xenograft volume reached about 100 mm^3^, mice were killed and the final weight of xenografts was recorded.

### Cell viability assay

Cells were precultured in 96-well plates (3 × 10^3^ per well) as triplicate wells and were allowed to adhere for 12 h. Following treatment (regarded as 0 h time point), cell viability was measured with CCK-8 kit (Dojindo laboratories, Tokyo, Japan) following the manufacturer’s instructions. Cells’ growth rate and sensitivity to chemical agents were calculated and formatted with GraphPad Prism 5.1.

### Apoptosis assay

Cells were digested with trypsin/EDTA (Gibco BRL) and resuspended with PBS. Annexin V-PE/7-AAD double staining was then performed for cells using an apoptosis detection kit (BD Biosciences, Erembodegem, Belgium) following the manufacturer’s instructions.

### Migration and invasion assay

150 μL resuspended cells (2 × 10^5^ per mL) were inoculated in each transwell (pre-coated with matrigel in terms of invasion assay) (Corning, New York, NY) upper chamber, then cultured in complete medium for 48 h. Transwells were fixed with methanol and dyed with 0.1% crystal violet after 48 h. Cells in upper chamber were removed with cotton wool, while penetrated cells were counted under 200 × microscope.

### Reagents and antibodies

Small interference RNAs (siRNAs) were from Ribobio (Guangzhou, China). Vectors overexpressing FLAG-tagged YARS or depleting YARS (shYARS), as well as related lentivirus were generated by GeneChem (Shanghai, China). Interference sequences for YARS were: sequence 1, 5′-ACTGAACAAGTTGCTGGAT-3′; sequence 2, 5′-CTGCACTTGGCTATTCAAA-3′.

Antibodies for YARS (ab150429, ab154819) were purchased from Abcam. Antibodies for p-S6 (S240/S244, #4858), p-S6(S240/S244, #5364), S6(#2217), p-Akt (S473, #9271), p-Akt (S473, #4060), Akt (#4691), p-Erk (T202/Y204, #4370), Erk(#4695), p-mTOR (Ser2448, #5536), mTOR (#2983), EGFR (#4267), FLAG-tag (#14,793), ATM (#92,356), BRCA1 (#14,823), MRE11 (#4847), 53BP1 (#88,439), RAD51 (#8875), GAPDH (#2118) were purchased from Cell Signaling technology. Fludarabine (S1491) and BEZ235 (S1009) were purchased from Selleck.

### Western blot assay

Cells were lysed using 1 × boiling SDS-PAGE loading buffer (1% SDS, 11% glycerol, 10% β-mercaptoethanol, 0.1 M Tris, pH 6.8) and collected for assay. For each sample, 20 μg total protein was applied in 20 μL volume for SDS-PAGE. Samples were probed with corresponding primary/secondary antibodies and the Clarity Western ECL substrate (Bio-Rad, Hercules, CA). Protein bands were visualized with Amersham Imager 600 (GE Healthcare, Chicago, IL).

### Immunohistochemistry assay

Formalin-fixed and paraffin-embedded (FFPE) tissue slides were deparaffinized with dimethylbenzene, rehydrated with ethanol, and treated with 3% H_2_O_2_ to quench endogenous peroxidase activity. Slides were boiled in sodium citrate buffer (10 mM, pH 6.0) for antigen retrieval and were incubated by 5% goat serum to block non-specific bindings. Primary and secondary antibodies were diluted with 5% goat serum to proper concentrations. Stained slides were independently examined and scored with light microscope (40 ×) by two pathologists from Peking University Cancer Hospital. The immunohistochemical score was determined based on the percentage and intensity of positive staining (nuclear or cytoplasmic in this study) in observed cells. A slide was classified into five percentage grade according to the percentage of stained cells in the section. (0, 1, 2, 3, 4 representing stained cells < 5%, 5–25%, 25–50%, 50–75%, > 75%, respectively), or classified into four intensity grades according to staining intensity (0, 1, 2, 3 representing null, weak, moderate and strong staining, respectively). The total immunohistochemical score of each specimen was defined as percentage grade × intensity grade. Specimens scored as < 1, 1–4, 5–8, > 9 were then further classified as −, +, ++, +++. For expression of a protein, −, + were determined as negative, ++, +++ as positive.

### Immunofluorescence staining

Cells were diluted to proper concentration (5 × 10^5^/mL) and pre-inoculated on coverslips 24 h before assessment. Cells were then treated with 4% paraformaldehyde for fixation and with 0.1% Triton X-100 for permeabilization at room temperature. Antigens were blocked by 5% goat serum, probed with primary antibody and stained with FITC-conjugated secondary antibody. Nuclei were stained with DAPI (1 μg/mL). Immunofluorescence images were observed and captured with the Zeiss LSM780 laser confocal microscope system.

## Results

### YARS was specifically upregulated in gastric cancer-derived specimens

**Fig. 1:**
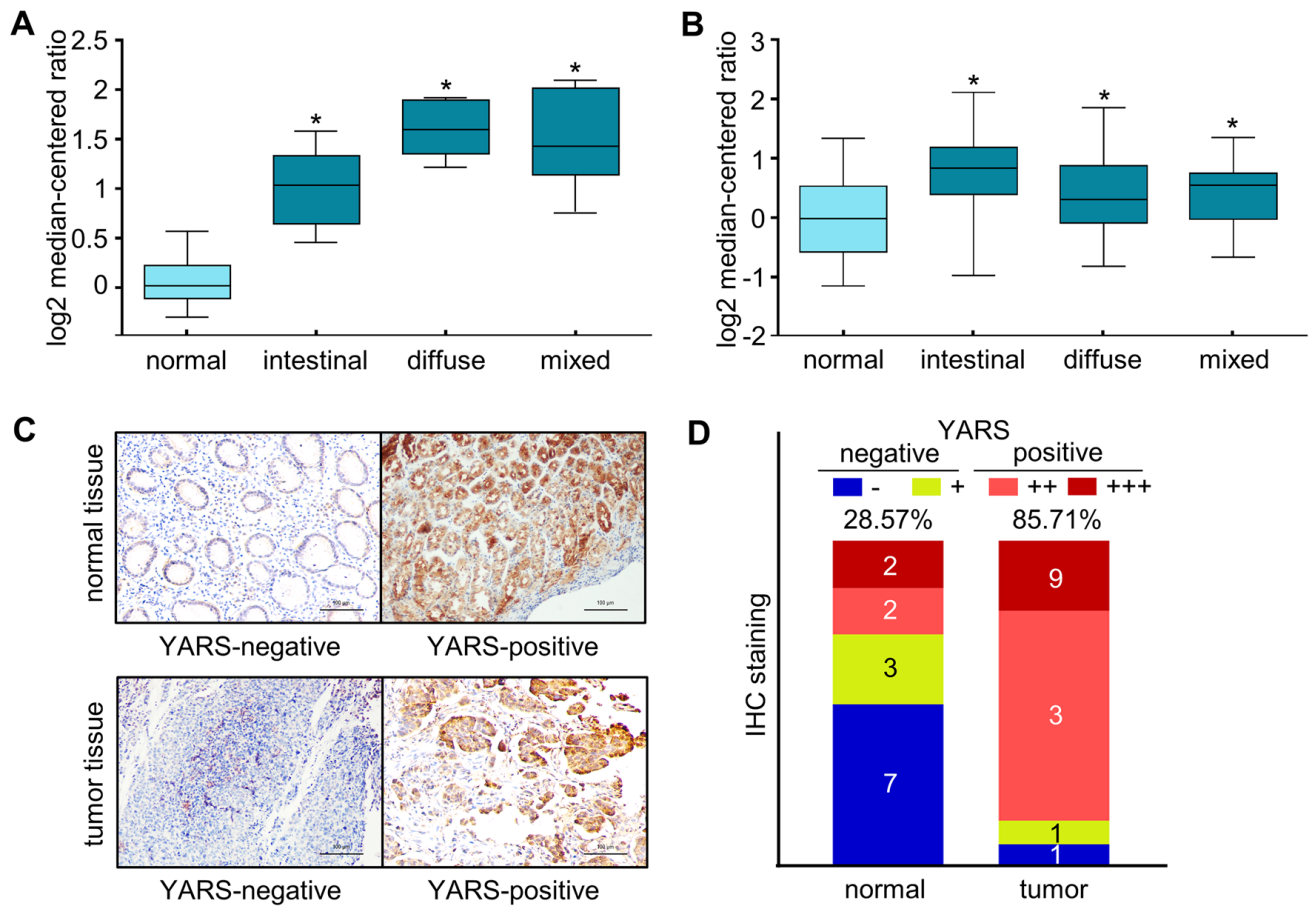
YARS was upregulated in gastric cancer-derived specimens**.** In GC datasets **a** GSE22377 and **b** TCGA-GC, transcript levels of YARS were compared between Lauren-classified tumor samples and normal samples. **c** Representative images of IHC staining for FFPE slides collected from 14 pairs of gastric cancer tissues or paired normal tissues. **d** IHC-based YARS expressional statistics for FFPE slides collected from 14 pairs of GC or paired normal tissues. *p < 0.05

YARS (tyrosyl RNA-synthetase) was originally recognized as a participant in mediating protein translation process. We first assessed the expressions of YARS in GC by analyzing public datasets. According to the Oncomine data portal (www.oncomine.org, originated from the study of Chen et al. 2003), transcript levels of YARS were consistently higher in intestinal, diffuse and mixed gastric tumor tissue than in nearby normal tissue (FC = 1.931, 2.931, 2.722, respectively) (Fig. 1a). By analyzing TCGA-GC data, we noticed that the transcript levels of YARS were also significantly higher in all gastric cancer Lauren subtypes than in normal tissues (FC = 1.652, 1.254, 1.268 for intestinal, diffuse and mixed subtypes vs. normal, respectively) (Fig. 1b).

We then further examined the expression of YARS between cancer and normal specimens. Immunohistochemistry staining in 14 pairs of surgery resected tissue specimens indicated that the positive rate of YARS protein expression was evidently higher in GC than in normal tissues (12/14, 85.71% vs. 4/14, 28.57%, Fig. 1c, d). These findings validated that YARS was specifically upregulated in gastric cancer-derived specimens, implicating its potential role in gastric cancer. Moreover, IHC images from YARS-positive gastric normal and tumor tissues suggested that YARS was mainly localized in both nuclei and cytoplasm (Supplementary Fig. S1A), which was also confirmed by immunofluorescence staining in GES-1, HGC-27, and AGS cells (Supplementary Fig. S1B).

### Expressional and mutational landscape of YARS in gastric cancer

**Fig. 2:**
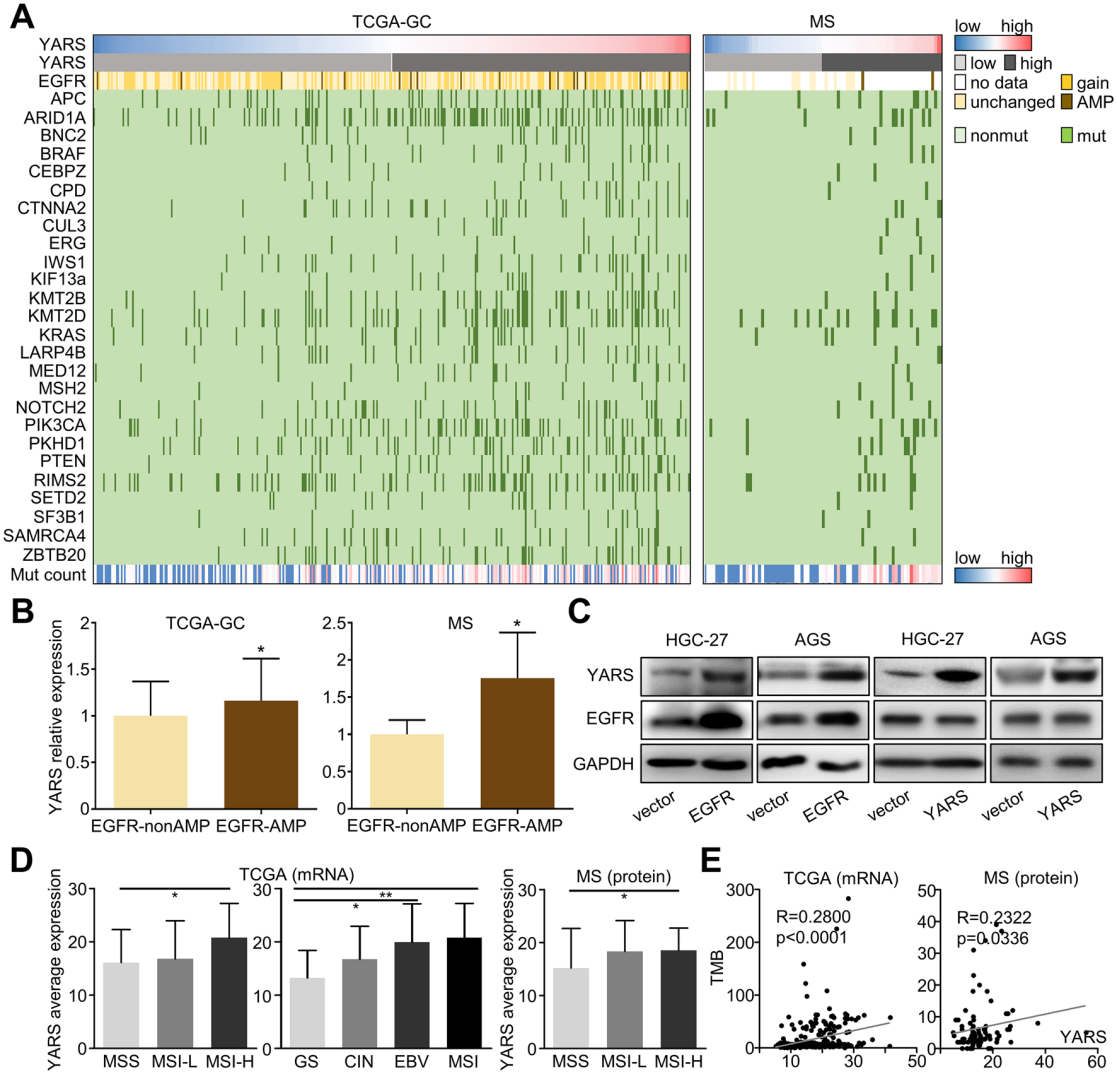
Expressional and mutational landscape of YARS in gastric cancer datasets. **a** The landscape of YARS expression, EGFR amplification, 26 other mutations, as well as the mutation burdens across gastric cancer TCGA-GC and MS datasets were displayed. **b** For TCGA-GC and MS datasets, relative expressions of YARS in EGFR-nonamplified (EGFR-nonAMP) and EGFR-amplified (EGFR-AMP) groups were compared. **c** Changes of YARS and EGFR in HGC-27/AGS cells after overexpressing YARS or EGFR were assessed by western blot. **d** For TCGA-GC and MS datasets, relative expressions of YARS among different molecular subtypes (MSS/MSI-L/MSI-H, or GS/CIN/EBV/MSI) were compared. **e** For TCGA-GC and MS datasets, the mutual correlation between YARS expression and TMB was compared with Pearson’s correlation analysis. *p < 0.05

We assessed the genomic and expressional distribution of YARS across TCGA-gastric cancer datasets and our MS dataset. The mutations of 26 malignancy-related genes (including APC, ARID1A, BNC2, BRAF, CEBPZ, CPD, CTNNA2, CUL3, ERG, IWS1, KIF13A, KMT2B, KMT2D, KRAS, LARP4B, MED12, MSH2, NOTCH2, PIK3CA, PKHD1, PTEN, RIMS2, SETD2, SF3B1, SMARCA4, ZBTB20) were found accumulated in patients harboring high-YARS transcript (TCGA-GC) or protein (MS) level (Fig. 2a). Specifically, we noticed that both mRNA and protein levels of YARS were higher in EGFR amplified than non-amplified patients (Fig. 2b). EGFR amplification was correlated with its high expression (Supplementary Fig. S2A) and stimulates the expression of downstream pathways/molecules through driving multiple transcription factors (Bhargava et al. 2005; Corcoran et al. 2012). For verification, we performed EGFR or YARS plasmid transfection in HGC-27/AGS cells. Overexpressing EGFR significantly upregulated YARS in both GC cell lines, while EGFR was unaffected by YARS upregulation, indicating that YARS was a downstream target of EGFR (Fig. 2c). By referring to the Animal Transcription Factor Database (https://bioinfo.life.hust.edu.cn/AnimalTFDB/), we noticed a series of EGFR’s canonical downstream transcription factors (such as STATs) were predicted to regulate YARS, among which STAT1 possessed the highest score (Supplementary Fig. S2B). In accordance with this prediction, expression of YARS protein in GC cells was impaired by the STAT1 inhibitor Fludarabine (Supplementary Fig. S2C). Therefore, we inferred that EGFR amplification or upregulation may promote YARS expression in a STAT1-involved transcriptional manner.

On the other hand, we also investigated YARS’s linkage with GC’s molecular subtypes. According to TCGA-GC cohort, expression of YARS transcript was higher in MSI-H (microsatellite instability-high) than in MSS (microsatellite Stable) patients or higher in MSI/EBV (Epstein-Barr virus) subtypes than in CIN (chromosomal instable)/GS (genomic stable) subtypes (Fig. 2d, left panel); while in MS data, YARS’s protein expression was also higher in MSI-H than in MSS patients (Fig. 2d, right panel). Furthermore, both protein and mRNA levels of YARS were positively correlated with TMB (tumor mutation burden) (Fig. 2e). These data suggested that YARS in GC might be upregulated by certain genetic changes (i.e., specific mutations or EGFR amplification) and YARS-high GC populations were largely overlapped with the patients suitable for immunotherapy (MSI-H or TMB-high) (Le et al. 2015).

### YARS promoted the malignant progression and was correlated with adverse prognosis in gastric cancer

**Fig. 3:**
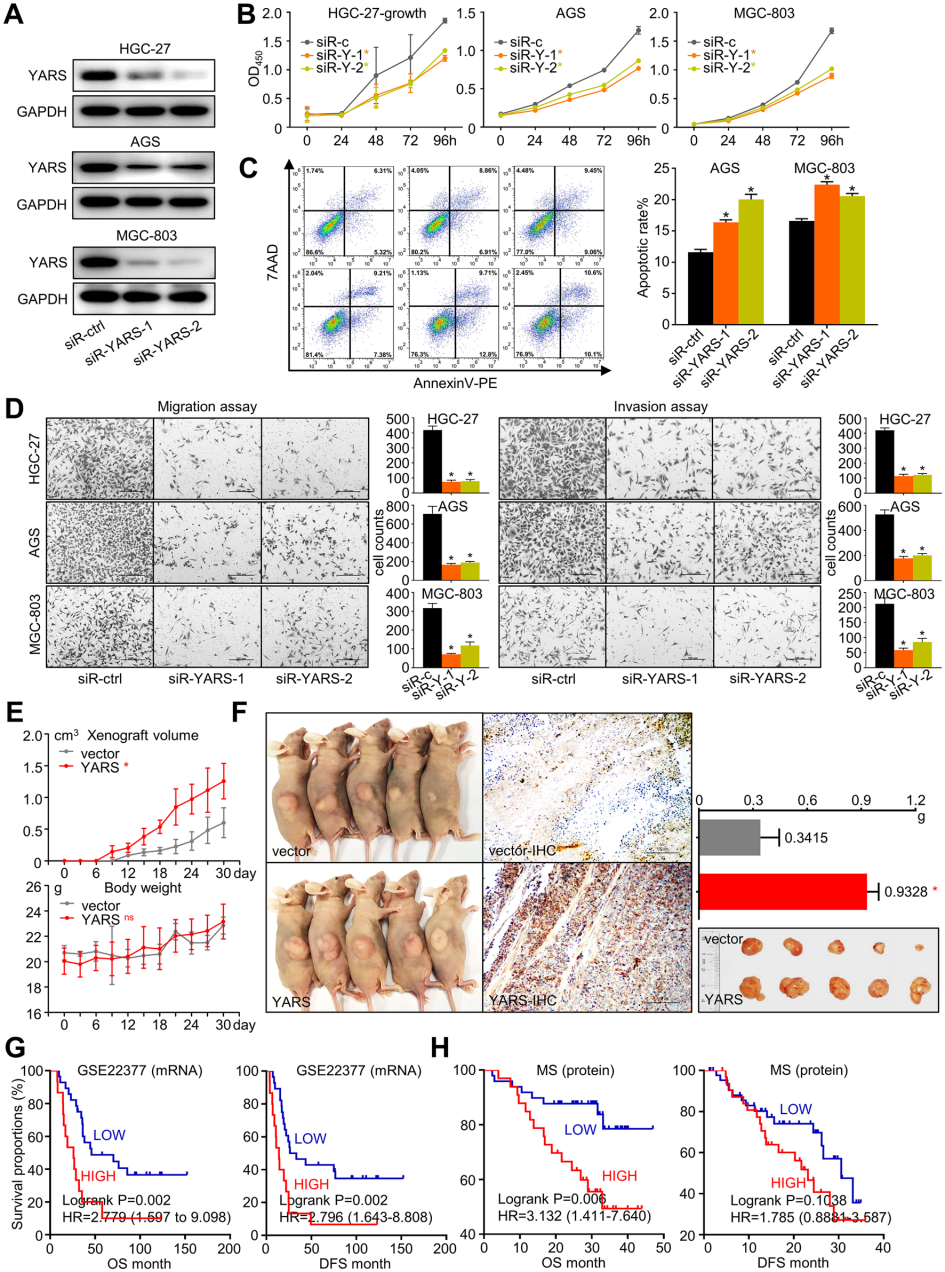
YARS promoted the malignant progression and was correlated with adverse prognosis in gastric cancer. For HGC-27 and AGS cells, changes of **a** YARS protein expressions, **b** cell proliferation, **c** apoptosis, and **d** migration/invasion after YARS being knocked down by siRNA. For HGC-27-derived xenograft models, **e** in vivo tumor growth, mice body mass changes, **f** xenograft size/weight and statistics were compared between HGC-27 and HGC-27-YARS groups. Overall survival (OS) and disease-free survival (DFS) of patients from **g** GSE22377 and **h** MS datasets were indicated by YARS stratifications. *p < 0.05. *ns* not significant

To decipher YARS’s impact on cancer progression, we assessed changes of multiple cancerous phenotypes after siRNA-based YARS knockdown (Fig. 3a). The proliferation rate of gastric cancer cell lines HGC-27, AGS, and MGC-803 significantly dropped after YARS interference (Fig. 3b). Furthermore, apoptotic rates were significantly enhanced by YARS interference (Fig. 3c), while migration/invasion capability was eliminated by YARS interference (Fig. 3d). We then constructed YARS stably overexpressed HGC-27 and AGS cell lines by performing lentivirus infection (Supplementary Fig. S3A). Accordingly, YARS overexpression significantly enhanced the in vivo tumor growth rate and weight in HGC-27-derived xenograft independent of the whole body mass changes (Fig. 3e, f).

We then investigated YARS’s prognostic relevance by analyzing omics data from multiple independent studies. Patients were classified into “high” and “low” groups according to their expressions of YARS mRNA/protein. On transcriptional level, although the diversity of OS (overall survival) between high/low YARS patients were subtle in the TCGA-GC cohort (*n* = 441, Supplementary Fig. S4), high-YARS was significantly paired with unfavorable prognosis in an expression profiling array-based study (GSE22377, *n* = 44) (Fig. 3g). On protein level, according to a mass spectrum-based proteomics study, we previously performed for GC patients (MS, *n* = 84), high-YARS expression also predicted an adverse overall survival (Fig. 3h). Taken together, these phenomena emphasized that YARS upregulation promoted the malignant progression of gastric cancer.

### YARS promoted gastric cancer progression through activating PI3K-Akt signaling

**Fig. 4:**
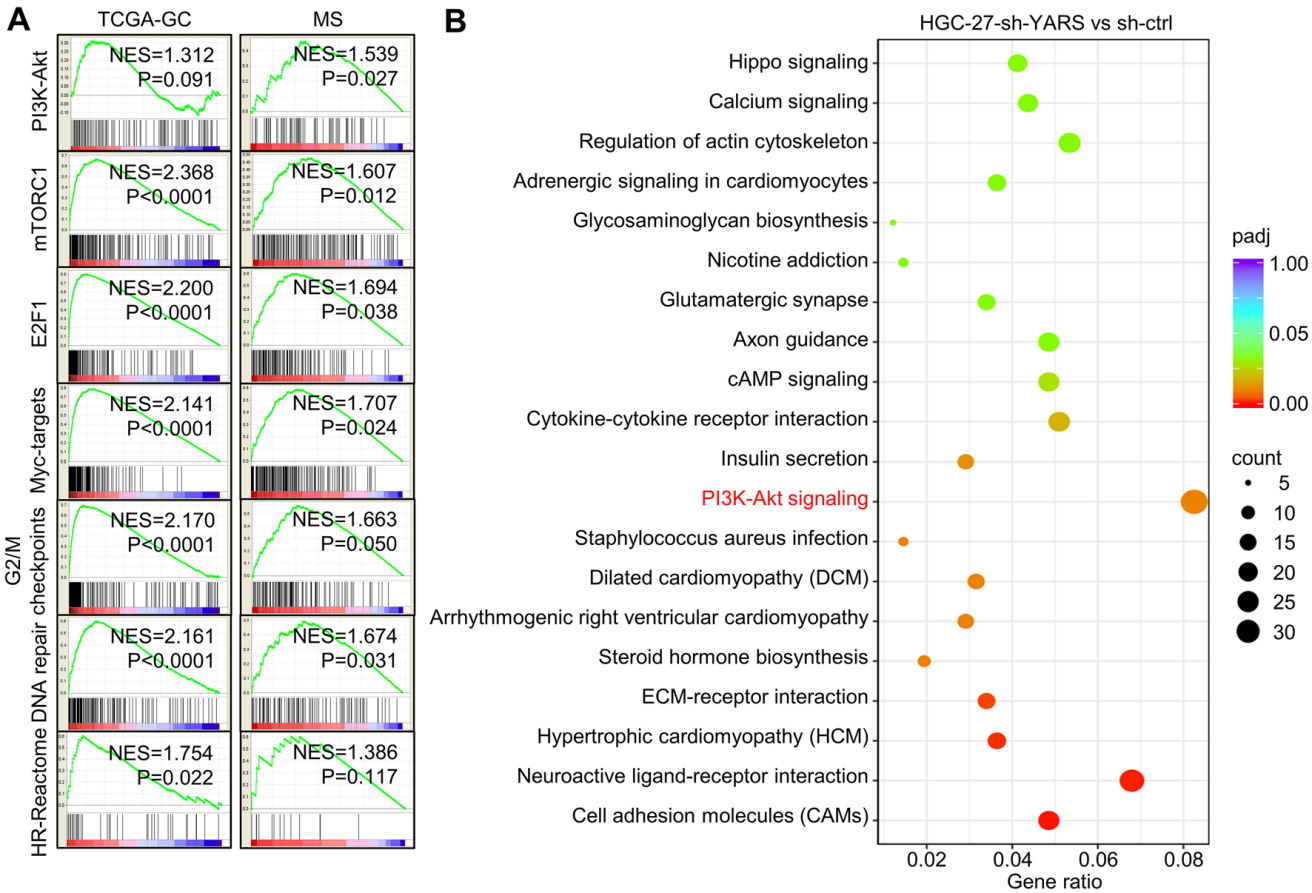
Functional prediction of YARS in gastric cancer. **a** For both TCGA-GC and MS datasets, the seven gene sets with the highest enrichment scores [PI3K-Akt, mTORC1, E2F1, Myc-targets, G2/M checkpoints, DNA repair and homologous recombination (HR)] were enriched according to YARS expression. NES, normalized enrichment score. **b** According to HGC-27-based RNA-sequencing, the top 20 pathways/processes enriched in after administered with shYARS were displayed

**Fig.5:**
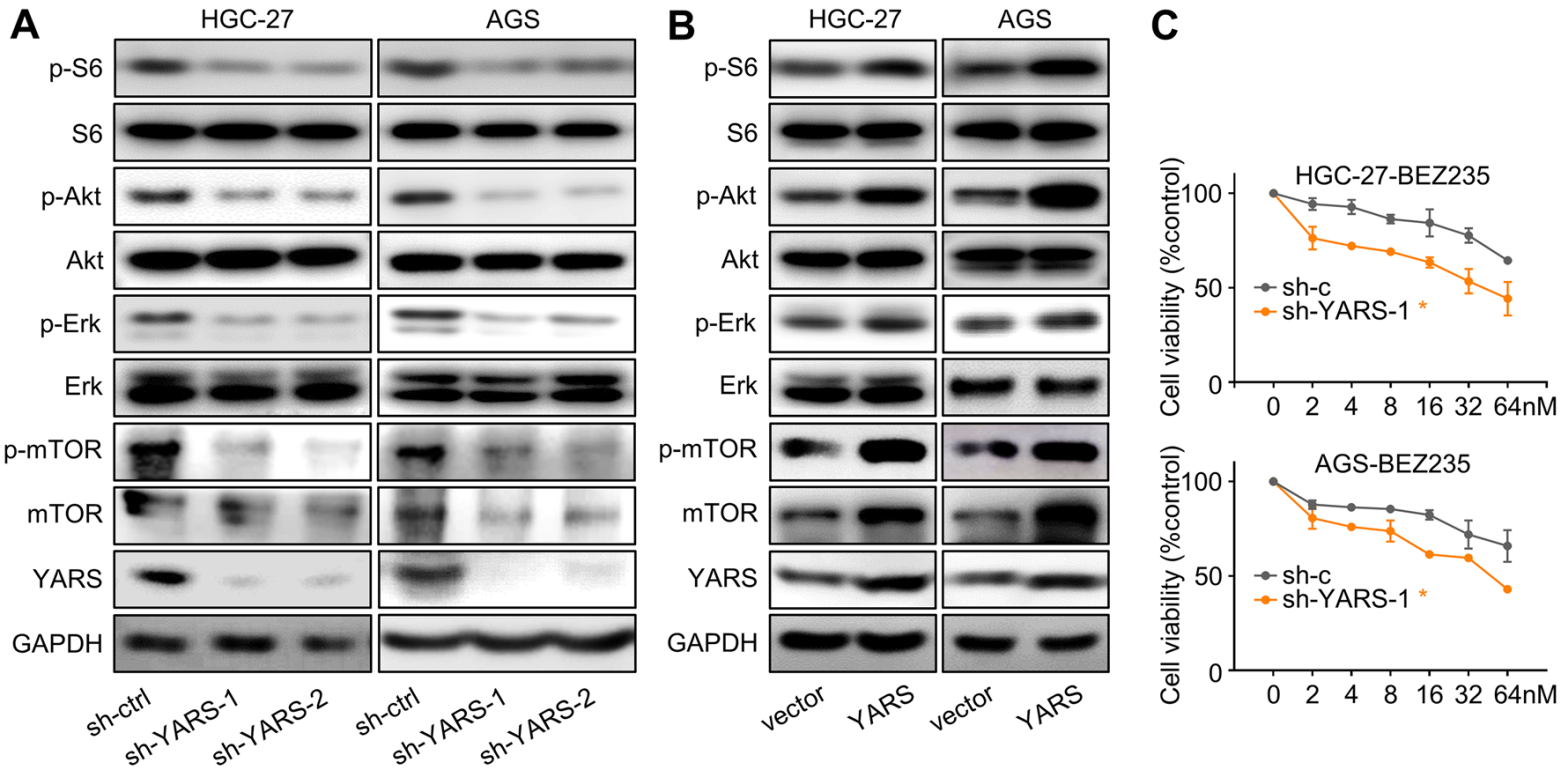
YARS activated PI3K-Akt signaling in gastric cancer. For HGC-27/AGS cells, changes of the PI3K-Akt pathway representative members (p-S6, S6, p-Akt, Akt, p-Erk, Erk, p-mTOR, mTOR) and YARS after **a** YARS depletion or **b** YARS overexpression were assessed by western blot. **c** After YARS depletion in GC cells, 48 h responses to the concentration cascades of the PI3K-Akt pathway inhibitor BEZ235 were assessed with CCK-8 assay. *v* vector, *Y* YARS. *p < 0.05

To decipher YARS-related mechanisms in promoting GC development, we classified patients into YARS-low and YARS-high groups on expressional level, then predicted its association with phenotypes and signaling pathways by performing Gene Set Enrichment Analysis (GSEA). For both TCGA-GC and MS datasets, PI3K-Akt, mTOR, E2F1, Myc, G2/M checkpoint, DNA repair, and homologous recombination related pathways/processes were enriched in YARS-high groups (Fig. 4a). We then carried out RNA-sequencing for HGC-27 accepting shYARS transfection and listed all the differentially expressed genes (DEGs) after YARS knockdown. After YARS knockdown, PI3K-Akt pathway and cell adhesion molecules (CAMs) were significantly enriched (Fig. 4b). For verification, we performed western blot analysis for several representative members of the PI3K-Akt pathway (p-S6, S6, p-Akt, Akt, p-Erk, Erk) in YARS interfered/overexpressed GC cells. The phosphorylation of these molecules was strongly repressed by depletion of (Fig. 5a) or enhanced by upregulation of YARS (Fig. 5b). Furthermore, according to IHC results, the levels of p-S6, p-Akt, and p-Erk were consistently higher in YARS-positive samples than in YARS-negative samples (Supplementary Fig. S3B). Therefore, YARS has a capacity to stimulate PI3K-Akt signaling.

On the other hand, YARS depletion (mediated by the representative interference sequence sh-YARS-1) significantly strengthened HGC-27/AGS cells’ sensitivity to the PI3K/mTOR inhibitor BEZ235 (Fig. 5c), which might be done through augmenting PI3K-Akt pathway inhibition, as well as silencing other growth-associated pathways downstream of YARS. Furthermore, the PI3K-Akt pathway activation (marked by S6 and Akt phosphorylation) induced by YARS overexpression was repressed after BEZ235 administration (Fig. 7a), while YARS enhanced cell proliferation (Fig. 7b) and invasiveness (Fig. 7c, d) were also rescued by BEZ235. These phenomena validated that YARS exerted its malignant roles in GC through activated PI3K-Akt signaling.

### YARS enhanced homologous recombination through activating PI3K-Akt signaling

**Fig. 6:**
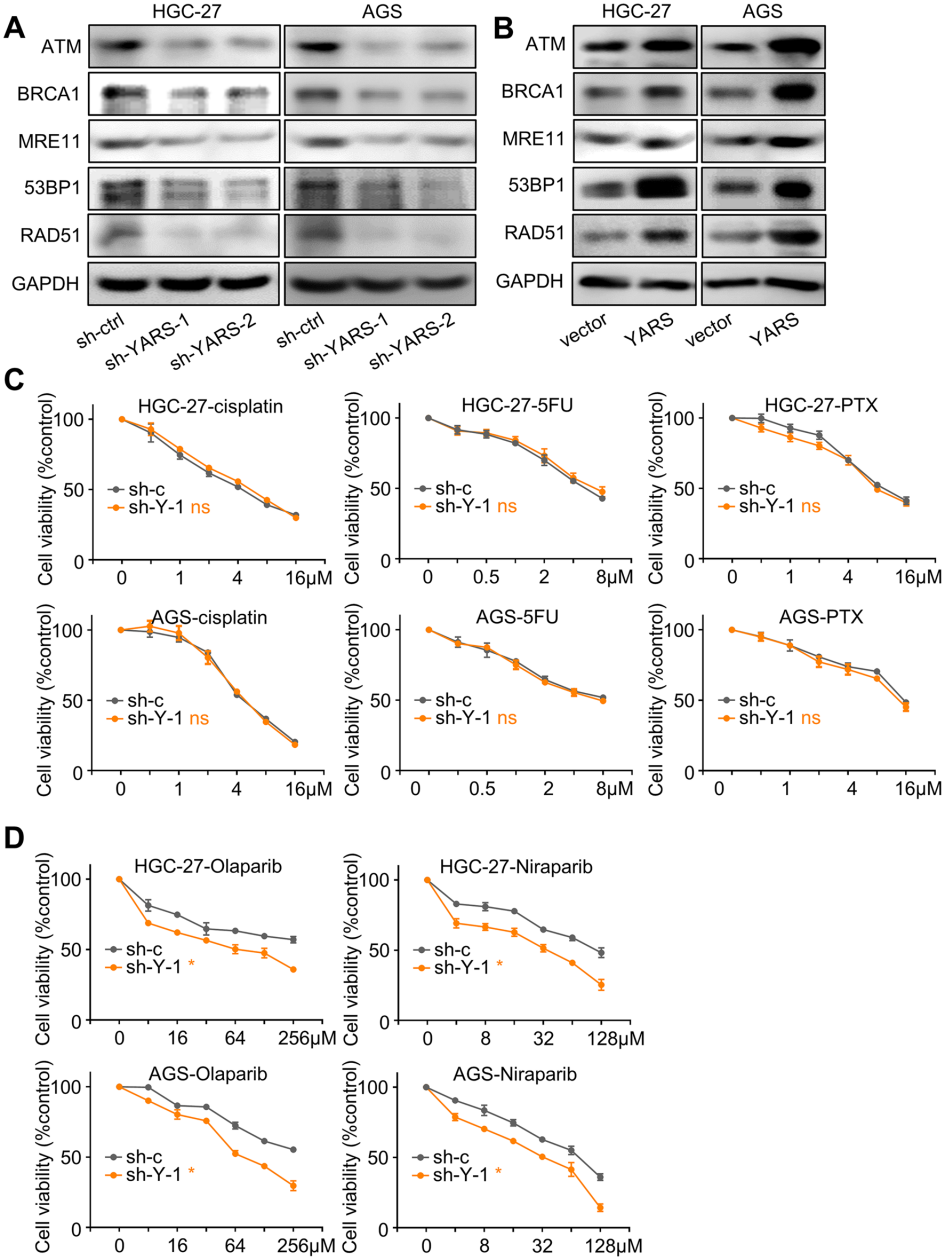
YARS promoted homologous recombination and insensitized responses to PARP inhibitors in gastric cancer. For HGC-27/AGS cells, changes of the homologous recombination pathway representative components (ATM, BRCA1, MRE11, 53BP1, RAD51) after **a** YARS depletion or **b** YARS overexpression were assessed by western blot. After YARS depletion in GC cells, 48 h responses to the concentration cascades of **c** three chemotherapy agents (cisplatin, 5-FU, paclitaxel) or **d** two PARP inhibitors (Olaparib, Niraparib) were assessed with CCK-8 assay. *v* vector, *Y* YARS. *p < 0.05. *ns* not significant

As previously demonstrated by GSEA in TCGA and MS datasets, high-YARS expression was correlated with DNA repair and homologous recombination (HR) processes. Thus, we investigated YARS’s impact on HR-related phenotypes. According to western blot analysis, levels of HR-related molecular markers (ATM, BRCA1, MRE11, 53BP1, RAD51) (Helleday 2016) in HGC-27 and AGS were consistently repressed by YARS depletion or enhanced by YARS upregulation (Fig. 6a, b). Since HR-defected tumors were anticipated to respond vigorously to PARP (Poly (ADP-ribose) polymerase) inhibitors (Hoppe et al. 2018), we simultaneously testified GC cells’ sensitivity to PARP inhibitors (Olaparib and Niraparib) after YARS knockdown. Although the sensitivity of GC cells to chemotherapy agents (cisplatin, 5-FU and paclitaxel) remained unaffected (Fig. 6c), sensitivity to Olaparib and Niraparib was strengthened by YARS depletion (Fig. 6d), suggesting that YARS enhanced homologous recombination hinders the efficacy of PARP inhibitors in GC.

**Fig. 7:**
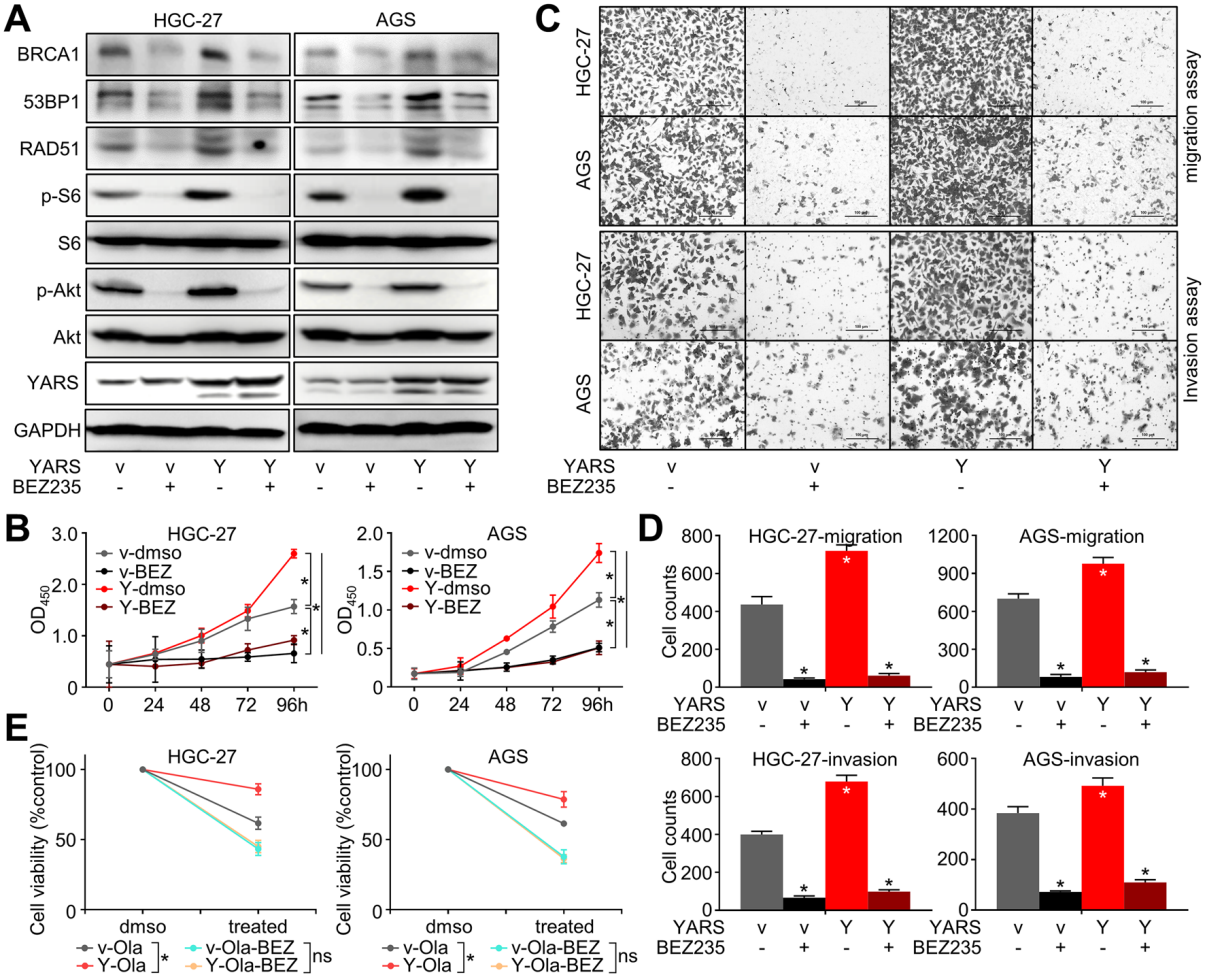
YARS augmented proliferation, invasiveness, and homologous recombination in gastric cancer through activating PI3K-Akt signaling. For YARS-overexpressed HGC-27/AGS cells, **a** changes of the PI3K-Akt pathway representative members (p-S6, S6, p-Akt, Akt), the homologous recombination pathway representative components (BRCA1, 53BP1, RAD51) and YARS; **b** changes of cell proliferation, as well as **c**, **d** changes of migration/invasion after BEZ235 administration were assessed, while **e** the 48 h responses to Olaparib combining BEZ235 were assessed with CCK-8 assay. *v* vector, *Y* YARS. *p < 0.05. *ns* not significant

Since the activation of PI3K-Akt has been reported to augment homologous recombination, we assessed whether YARS-enhanced HR depends on P3K-Akt signaling. Upregulation of HR-related molecules (BRCA1, 53BP1, RAD51) induced by YARS overexpression was repressed by BEZ235 treatment (Fig. 7a). We then treated GC cells with Olaparib (64 μM) alone or a combination of Olaparib with BEZ235 (32 nM). GC cells’ sensitivity to Olaparib was impaired by YARS overexpression, while this repression was rescued by introduction of BEZ235 (Fig. 7e). These phenomena hinted that through activating PI3K-Akt signaling, YARS enhanced homologous recombination and impaired GC’s sensitivity to PARP inhibitors.

## Discussion

As a housekeeping gene that facilitates tyrosyl aminoacylation, YARS is traditionally considered playing a fundamental role in maintaining basic biological activities. To date, although mutations of YARS were reported correlated with neuropathy or development disorders, YARS’s association with cancer has not yet been characterized. In this study, we explored YARS’s functions in gastric cancer by applying bioinformatics analysis and wet lab experiments. We discovered that both YARS transcript and protein were highly expressed in GC specimens, which was correlated with poor prognosis. Through analyzing GC datasets, we revealed YARS’s co-expression with EGFR amplification, specific mutations, tumor mutation burden, EBV/MSI phenotypes, as well as multiple gene sets potentially those were enriched by YARS high expression. By referring to cell line-based RNA-sequencing, we focused on PI3K-Akt signaling and further validated its association with YARS. YARS elicited multiple malignant phenotypes through activating PI3K-Akt signaling, while the YARS-induced homologous recombination and insensitivity to PARP inhibitors also depended on PI3K-Akt. To our knowledge, it is the first report to unveil the malignant roles and potential applications of aminoacyl-tRNA synthetase in cancer.

As discovered in patients carrying CMT neuropathy, missense mutations and deletions of YARS (such as Gly41Arg, Asp81Ile, Glu196Lys, Glu196Gln and 153_156del) lead to a loss of its aminoacylation activity and subsequently reduced cell growth (Gonzaga-Jauregui et al. 2015; Jordanova et al. 2006; Schabhuttl et al. 2014). Apart from mutations of YARS, loss-of-function variants of other types of aaRS such as AARS (alanyl-tRNA synthetase), HARS (histidyl-tRNA synthetase), KARS (lysyl-tRNA synthetase), and MARS (methionyl-tRNA synthetase) were also reported to be related with Charcot–Marie–Tooth disease and other types of neuropathy (Abbott et al. 2018; Gonzalez et al. 2013; McLaughlin et al. 2010, 2012; Vester et al. 2013). Interestingly, despite the fact that aaRSs were basic participants of aminoacylation and protein synthesis as suggested by limited functional studies in yeast/worm/Drosophila models, aaRS mutation-related neuropathogenesis was more likely to be caused by inducing neuronal toxicity and synaptic degeneration, rather than by impaired canonical aminoacylation activity. Nevertheless, the mutation (10/440, 2.27%) and amplification (6/440, 1.36%) of YARS were relatively rare in gastric cancer, hence our study majorly focuses on the malignant function of YARS’s expressional changes. Because our limited cases of IHC indicated that YARS was positively expressed in both gastric normal and tumor tissues, the threshold of positiveness should be specified for potential clinical applications.

As a crucial signaling node stimulating multiple pathways and transcriptional factors (such as PI3K-Akt, RAS-RAF-MEK-ERK, PLC-γ, Src, and JAK-STAT), EGFR (epidermal growth factor receptor) was prevalently mutated in lung cancer, while its genetic amplification and overexpression were also major causative events for gastrointestinal cancers (Du and Lovly 2018; Yarden and Sliwkowski 2001). EGFR-targeted regimens (tyrosine kinase inhibitors and antibodies) have been proved as effective therapeutic options against lung cancer and colorectal cancer, while also displaying prospective applications in gastric cancer (Liu et al. 2018). We discovered that YARS was highly expressed in EGFR-amplified patients. Overexpressing EGFR upregulated, while repressing STAT1 downregulated the protein level of cellular YARS, giving hint that EGFR might induce YARS expression through stimulating its downstream transcription factors (such as STAT1). Yet, the specific regulatory mechanisms demand further exploration and whether EGFR-mediated YARS upregulation influences the sensitivity or resistance to EGFR-targeted therapy remains to be answered.

Our work exhibited that PI3K-Akt was an intermediate pathway for YARS-induced phenotypes. In addition to PI3K-Akt, we demonstrated in GC cells that YARS enhanced homologous recombination (HR), a major pathway that repairs DNA double strand breaks (DSBs) in mammalian cells (Scully et al. 2019). Genomic instability and DNA breaks are representative traits in carcinogenesis and malignant progression. Since these highly genotoxic events commit cells to apoptosis, multiple mechanisms are vigorously activated in cancer to repair DNA lesions and to maintain cancer survival (O'Kane et al. 2017). Consequently, inhibitors targeting PARP (poly (ADP-ribose) polymerase) are applied as anticancer agents by disrupting PARP-mediated DNA single strand breaks (SSBs) repair. Due to that DNA SSBs can be converted to DSBs, PARP inhibitors induce a synthetic lethality and achieve a hypersensitivity in patients harboring HR deficiency (such as deletions, loss-of-function mutations or low expressions of BRCA1/2, ATM, RAD51 and other components of HR machinery) or simultaneously accepting HR inhibition (Hoppe et al. 2018; Noordermeer and van Attikum 2019). We demonstrated that depleting YARS enhanced GC cells’ sensitivity to PARP inhibitors, while YARS-directed HR and impaired PARP-i sensitivity relies on the activation of PI3K-Akt signaling. Thus, it is reasonable to believe that besides combing PI3K-Akt inhibitors (Konstantinopoulos et al. 2019), PARP-targeted drugs combining YARS inhibitors might also be a potential therapeutic option against GC.

Several studies have also addressed aaRS’s association with regulative pathways and processes. A predictive work raised the potential interaction between aaRSs (including LRS, DRS, RRS, IRS, KRS, QRS, EPRS) and MAPK/PI3K-Akt pathways (Hyeon et al. 2019). YARS was reported directly interacting with TRIM28/HDAC1 and sequestering HDAC1-mediated deacetylation on E2F1, thus enhances E2F1-mediated upregulation of homologous recombination (HR) factors and provides protection against DNA damage (Wei et al. 2014). On the other hand, aaRSs were suggested as essential mediators of Myc-directed growth control (Zirin et al. 2019), which was in concert with our findings. Besides PI3K-Akt related (PI3K-Akt/mTOR), E2F1, HR/DNA repair and Myc-targets, G2/M checkpoints associated signaling was also predicted to be positively enriched in YARS high-expression groups. Hence, YARS’s association with cell cycle control and other additional carcinogenic pathways also deserves further investigation. Besides YARS, the upregulation of several aminoacyl-tRNA synthetases has also been observed in GC and multiple other types of malignancy (Hu et al. 2013), which might be due to the increased carcinogenic metabolism and requirements for protein translation. Although we have demonstrated that YARS induced multiple signaling activation and progression of GC, the malignant roles of other aaRSs were unexplored. Also, it remains unclear whether YARS exerted its malignant functions through canonically enhancing expressions of relevant pathway members or through non-canonical routes beyond aminoacylation. Notably, our data in HGC-27 and AGS cells showed that YARS positively regulated S6, Akt, Erk, and mTOR phosphorylation instead of protein expression, while directly lifted protein levels of HR-related molecules. Considering no evidences supported the notion that aaRSs harboring kinase activity, we inferred that YARS’s impact on phosphorylation might be done through modulating the upstream kinase/phosphatases of PI3K-Akt pathway, yet the mechanistic details remain to be elucidated.

In contrast to bacterial or low-eukaryotic homologs, high-eukaryotic YARS (including human) specifically contains a C-terminal domain and can be split by proteolysis into two distinct cytokine mimics: an IL-8 (interleukin 8)-like N-domain derivative and an EMAP II (endothelial-monocyte-activating polypeptide II)-like C-domain derivative (Wakasugi and Schimmel 1999). IL-8 causes the recruitment of inflammatory cells and is also implicated in mediating cancerous progression (Chao et al. 2019). By acting on the CXCR1/2 receptor, the IL-8-like fragment (aka “mini-YARS”) induces cell migration as a monomer or inhibits migration as a dimer (Vo et al. 2011); mini-YARS was also reported potentially leading to angiogenesis by activating VEGFR2 or VEGF (Tzima et al. 2005; Zeng et al. 2014). IL-8 has been characterized to activate PI3K-Akt signaling through modulating phosphorylation/activation of Akt and S6 (MacManus et al. 2007), while PI3K-Akt-mTOR signaling was also reported to enhance IL-8 production as feedback (Lin et al. 2019), which comply with our findings in gastric cancer. On the contrary to IL-8, EMAP II is a pro-inflammatory cytokine that exerts anti-endothelial and anti-angiogenic activities through binding to VEGF receptors and disrupting fibronectin and VEGF signaling (Awasthi et al. 2013). As a consequence, YARS’s role in controlling malignant progression might be related with its cytokine-releasing capabilities. Nonetheless, we observed in GC datasets that IL-8-related signaling was insignificantly correlated with YARS, while angiogenesis-related signaling was negatively enriched in patients harboring high-YARS level (Supplementary Fig. S5). These might be due to that the actual levels of YARS-derived mimics could not be appropriately represented by value of total-YARS generated by RNA-sequencing or proteomics study or due to the functional diversities between YARS-derived cytokine mimics and authentic IL-8 or EMAP II. Since no antibodies targeting mini-YARS or EMAP II-like domains were commercially applicable, the production, release, functional details, as well as the respective proportions of YARS-derived IL-8 and EMAP II mimics in GC remained unexplored in this study. Additionally, since the production of pro-inflammatory cytokines (i.e., IL-8, IL-6 and TNF-α) is known to be mediated by NF-κB signaling (Tak and Firestein 2001), whether NF-κB promotes YARS cleavage and mini-YARS release also deserved future investigation.

Furthermore, YARS displayed a trend of high expression in high-TMB, MSI-H, and EBV subtypes of GC. Since high-TMB, MSI, and EBV patients have been proved more likely to benefit from immunotherapy (Le et al. 2015), the linkage between YARS, microenvironment, and cancer immune responses merits future investigation. Considering that alterations of DNA damage repair pathway result in increased TMB and neoantigen loads (Park et al. 2019; Wang et al. 2018), we hypothesized that YARS-mediated HR upregulation might serve as a feedback event of mutations or viruses infections.

In conclusion, our work demonstrated that YARS functions in gastric cancer beyond a fundamental gene. Through activating PI3K-Akt signaling, YARS promotes malignant progression and insensitizes PARP inhibitors. Our work shed light upon the novel functions of housekeeping proteins, and also proposed the carcinogenic involvement and druggable applications of the aminoacyl-tRNA synthetase family.

## Electronic supplementary material

Below is the link to the electronic supplementary material.

**Figure S1. YARS was localized in both cytoplasm and nucleus of normal and tumor cell lines/tissues.(A) **The localization of YARS protein in gastric cancer/paired normal tissues harboring positive YARS IHC staining.** (B) **The localization of YARS protein in GES-1, HGC-27, and AGS cells. YARS was detected by primary and FITC-conjugated secondary antibodies. (TIF 6082 kb)

**Figure S2. EGFR induced YARS upregulation potentially in a transcription factor-associated manner. (A) **The relative expression of EGFR was significantly higher in EGFR amplified than in non-amplified GC patients. **(B)** The potential transcription factors for YARS predicted by the Animal Transcription Factor Database. The probability for the candidates as transcription factors of YARS was indicted by high predict scores, as well as *p*<0.05. **(C)** After treating with Fludarabine (100 μM for 36 h), the protein level of YARS in HGC-27 and AGS was assessed by western blot *, *p*<0.05. (TIF 714 kb)

**Figure S3. Establishment of YARS stably overexpressed GC cell lines, as well as YARS-associated IHC staining of PI3K-Akt signaling molecules in GC tissue. (A)** For HGC-27 and AGS cell lines stably overexpressed YARS, changes of FLAG-tag and YARS were measured by western blot. **(B)** For GC tissues, IHC staining of YARS, p-S6, p-Akt and p-Erk were performed for sequential slides. Displayed were the representative images for YARS-negative/positive tumor tissue. (TIF 22301 kb)

**Figure S4. Prognostic correlation of YARS in TCGA-GC dataset. **Overall survival (OS) and disease-free survival (DFS) of patients from TCGA-GC dataset were indicated by YARS stratifications. (TIF 241 kb)

**Figure S5. Enrichment for IL-8- and angiogenesis-related gene sets. **For TCGA-GC and MS datasets, Gene Set Enrichment Analysis was performed according to YARS expression. NES, normalized enrichment score. (TIF 1041 kb)
