# Supplementary figures and images for: YARS as an oncogenic protein that promotes gastric cancer progression through activating PI3K-Akt signaling
Source: J Cancer Res Clin Oncol. 2020 Jan 8;146(2):329–42. doi: 10.1007/s00432-019-03115-7 (PMC6985085; doi:10.1007/s00432-019-03115-7)

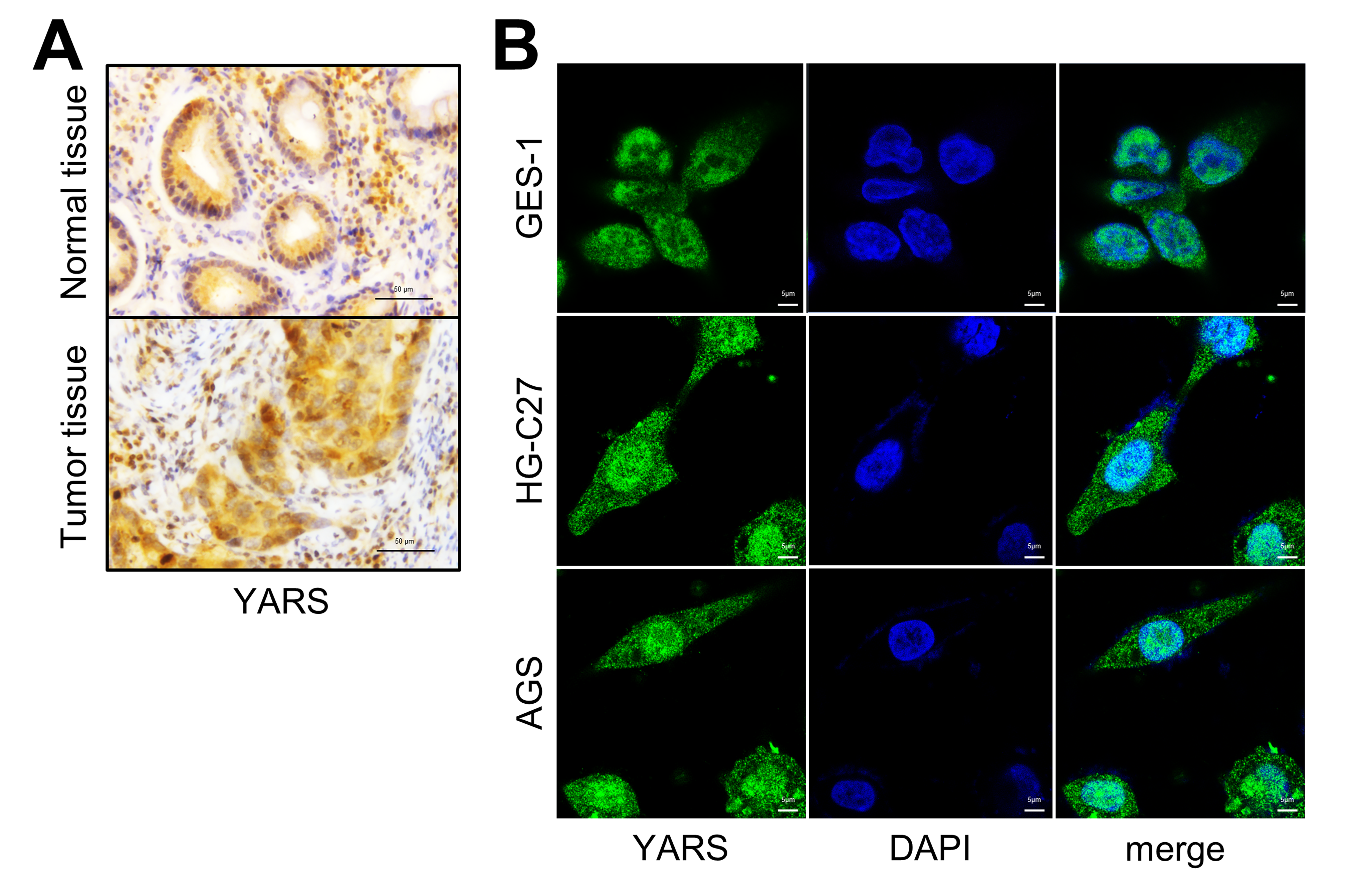

Supplement: Supplementary file 1 — Figure S1. YARS was localized in both cytoplasm and nucleus of normal and tumor cell lines/tissues.(A) The localization of YARS protein in gastric cancer/paired normal tissues harboring positive YARS IHC staining. (B) The localization of YARS protein in GES-1, HGC-27, and AGS cells. YARS was detected by primary and FITC-conjugated secondary antibodies. (TIF 6082 kb) [file 432_2019_3115_MOESM1_ESM.tif]

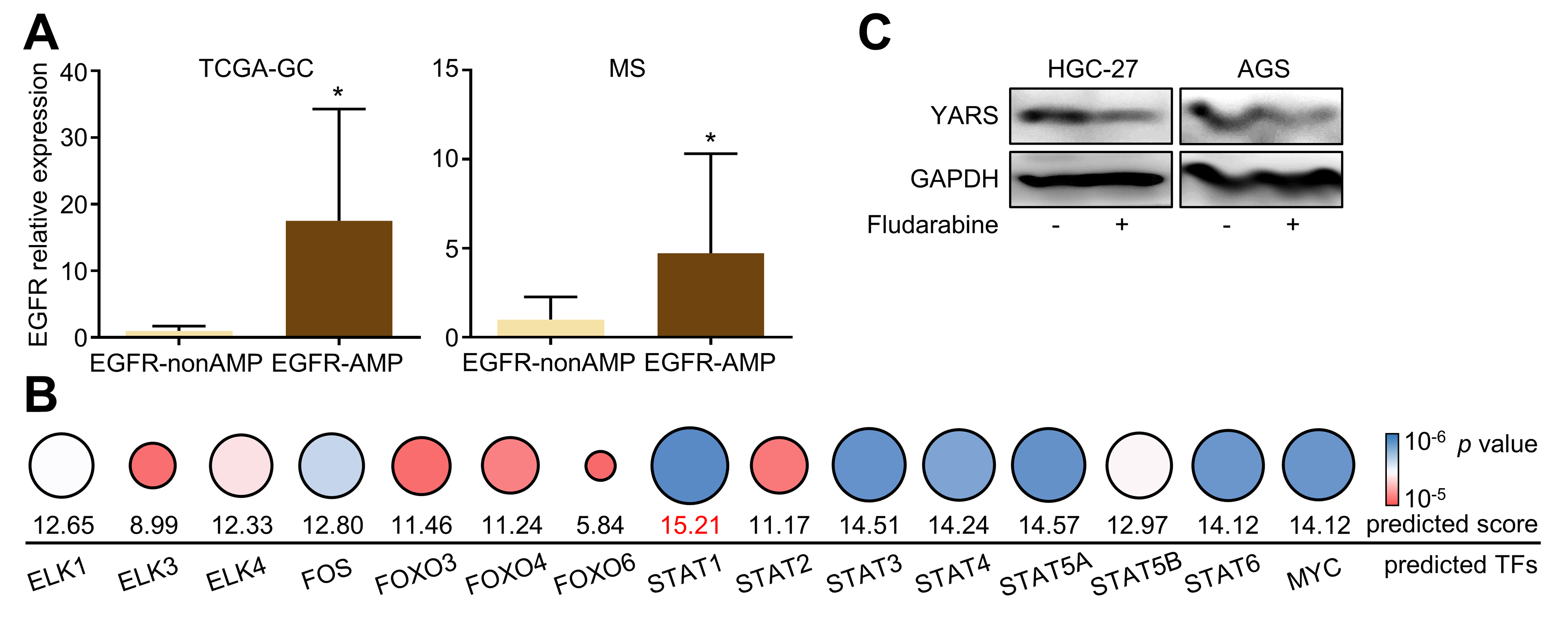

Supplement: Supplementary file 2 — Figure S2. EGFR induced YARS upregulation potentially in a transcription factor-associated manner. (A) The relative expression of EGFR was significantly higher in EGFR amplified than in non-amplified GC patients. (B) The potential transcription factors for YARS predicted by the Animal Transcription Factor Database. The probability for the candidates as transcription factors of YARS was indicted by high predict scores, as well as p<0.05. (C) After treating with Fludarabine (100 μM for 36 h), the protein level of YARS in HGC-27 and AGS was assessed by western blot *, p<0.05. (TIF 714 kb) [file 432_2019_3115_MOESM2_ESM.tif]

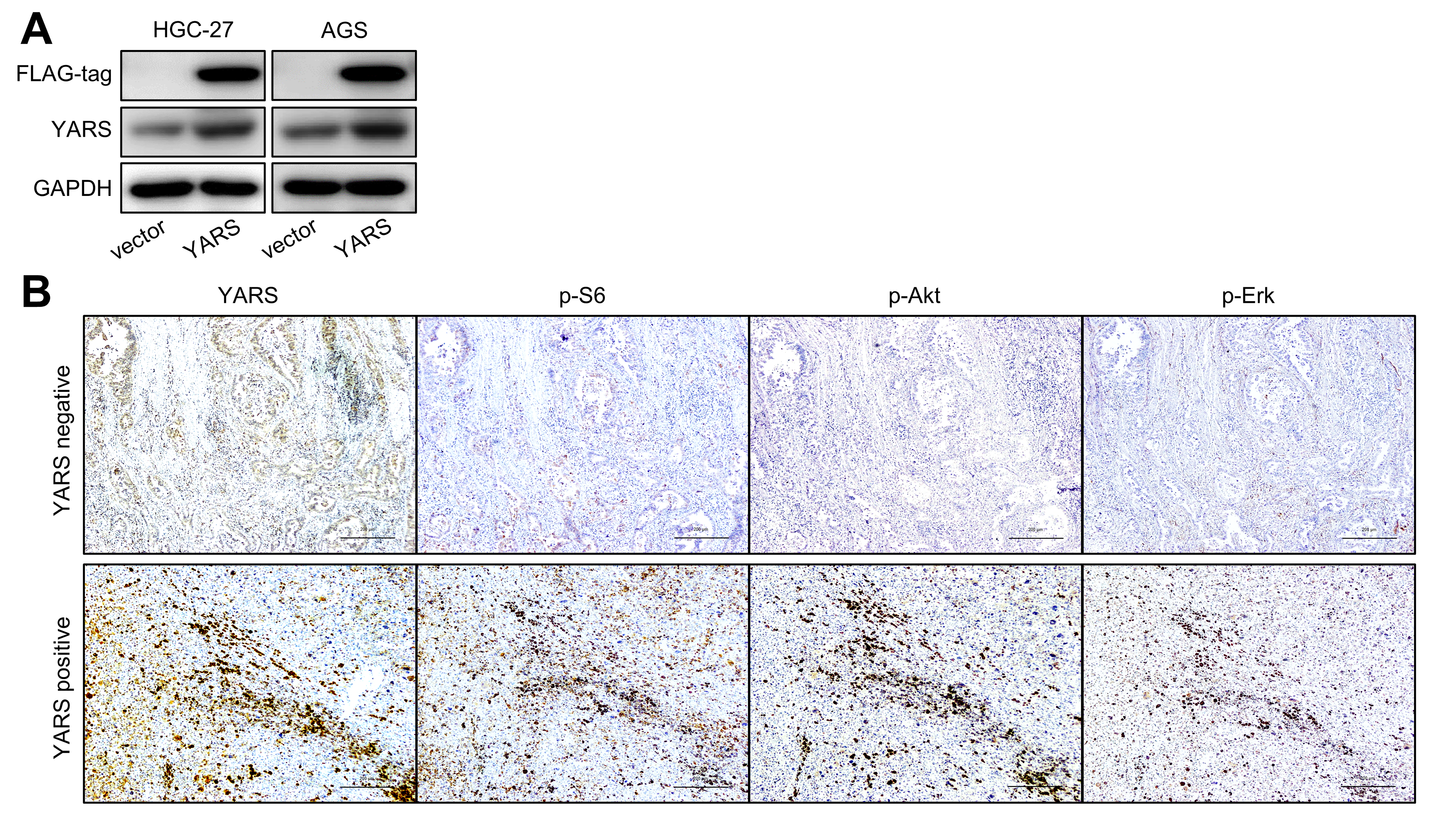

Supplement: Supplementary file 3 — Figure S3. Establishment of YARS stably overexpressed GC cell lines, as well as YARS-associated IHC staining of PI3K-Akt signaling molecules in GC tissue. (A) For HGC-27 and AGS cell lines stably overexpressed YARS, changes of FLAG-tag and YARS were measured by western blot. (B) For GC tissues, IHC staining of YARS, p-S6, p-Akt and p-Erk were performed for sequential slides. Displayed were the representative images for YARS-negative/positive tumor tissue. (TIF 22301 kb) [file 432_2019_3115_MOESM3_ESM.tif]

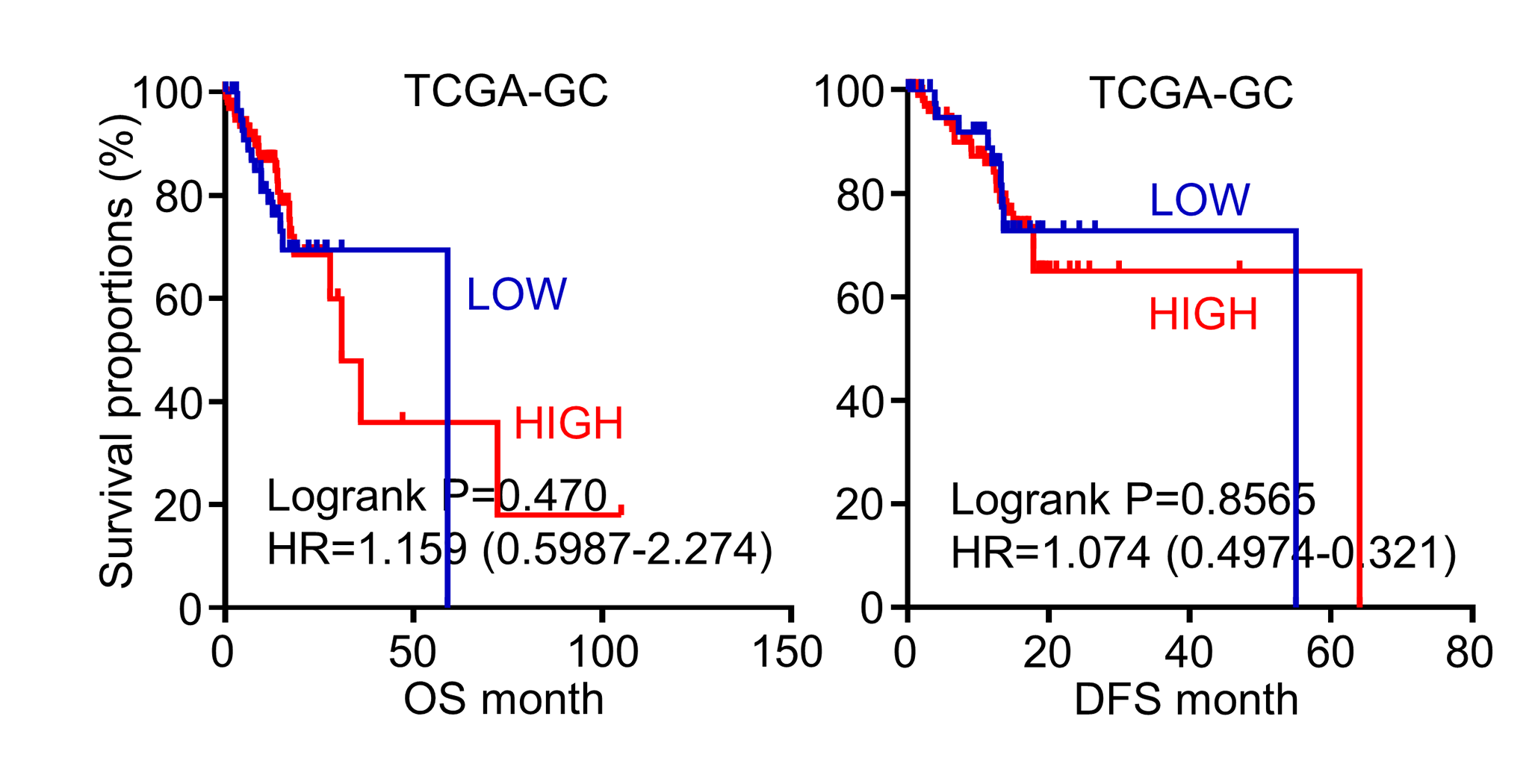

Supplement: Supplementary file 4 — Figure S4. Prognostic correlation of YARS in TCGA-GC dataset. Overall survival (OS) and disease-free survival (DFS) of patients from TCGA-GC dataset were indicated by YARS stratifications. (TIF 241 kb) [file 432_2019_3115_MOESM4_ESM.tif]

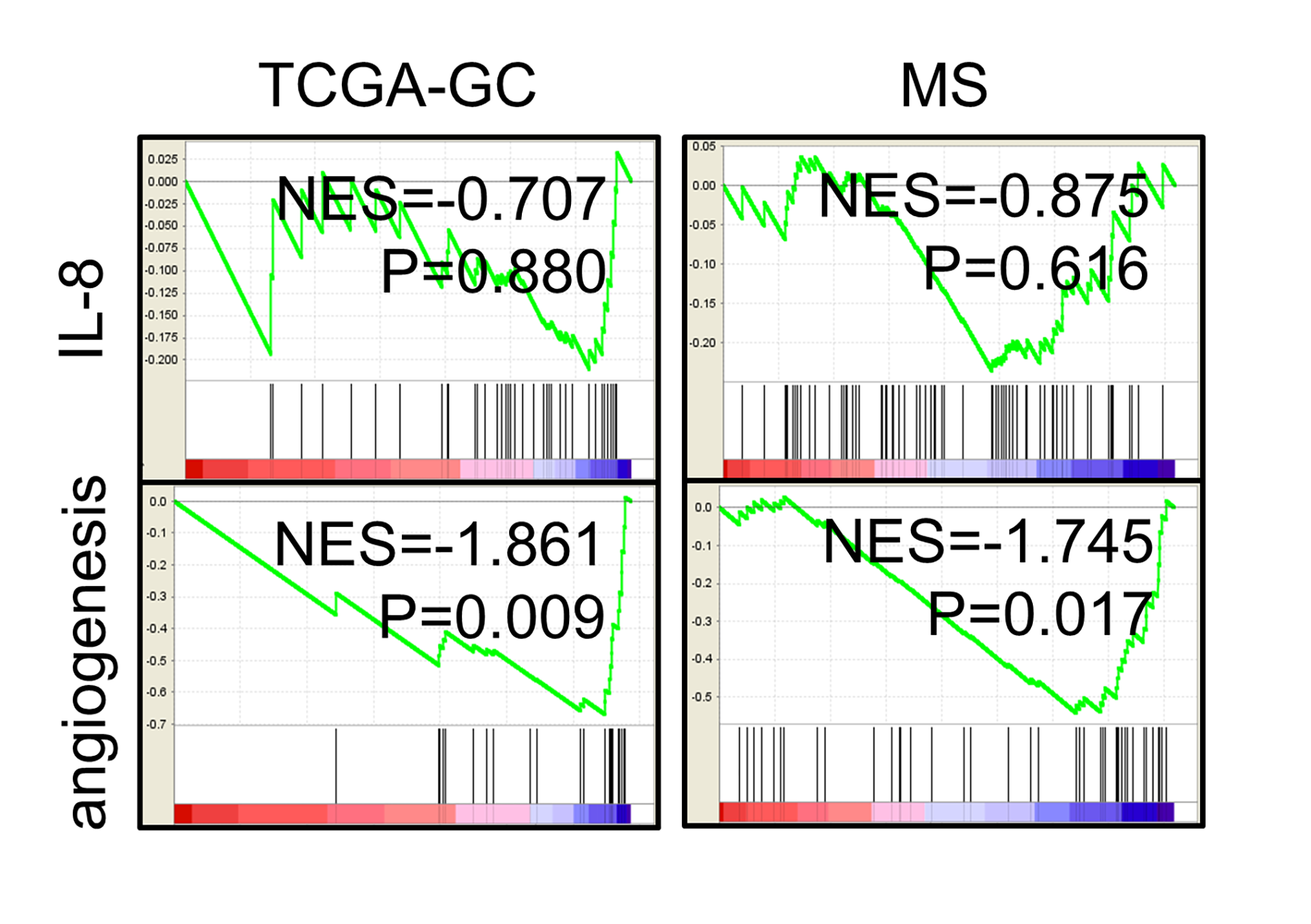

Supplement: Supplementary file 5 — Figure S5. Enrichment for IL-8- and angiogenesis-related gene sets. For TCGA-GC and MS datasets, Gene Set Enrichment Analysis was performed according to YARS expression. NES, normalized enrichment score. (TIF 1041 kb) [file 432_2019_3115_MOESM5_ESM.tif]
